# Orthodontic treatment induces Th17/Treg cells to regulate tooth movement in rats with periodontitis

**DOI:** 10.22038/ijbms.2020.44437.10419

**Published:** 2020-10

**Authors:** Nan Ge, Jing Peng, Lan Yu, Shuo Huang, Lu Xu, Ying Su, Li Chen

**Affiliations:** 1 Department of Orthodontics, Beijing Stomatological Hospital & School of Stomatology, Capital Medical University, Beijing 100050, China; 2 Department of Stomatology, Beijing Pinggu Hospital, Beijing 101200, China; 3 Department of Orthodontics, Stomatological Hospital of Guangzhou Medical University, Guangzhou 100079, China; 4 Department of Stomatology, Tie Ying Hospital of Fengtai District Beijing, Beijing 100079, China; 5 Institute of Dental Research, Beijing Stomatological Hospital & School of Stomatology, Capital Medical University, Beijing 100050, China

**Keywords:** Orthodontics, Osteoclasts, Periodontitis, Th17 cells, Treg cells

## Abstract

**Objective(s):**

Here we investigated the regulation of Th17 and Treg cells in orthodontic tooth movement during periodontal inflammation.

**Materials and Methods:**

Fifty-six SD rats were divided into a control (24 rats) and a tooth movement group during the recovery stage of periodontitis (RM group, 32 rats). Periodontitis was established by silk ligation and local injection of LPS. Orthodontic tooth movement was achieved by nickel-titanium springs on the maxillary first molars. The proportions of Th17 cells and Treg cells were evaluated by flow cytometry. Gene expression of ROR-γt and Foxp3 was determined by real-time PCR. Expression of ROR-γt, Foxp3, RANK, RANKL, and OPG was detected by immunohistochemical staining. Osteoclasts were detected by TRAP staining. Relationships between Th17/Treg cells, osteoclasts, and related factors were estimated by correlation and regression analysis.

**Results:**

During orthodontic tooth movement in the recovery stage of periodontitis, the proportion of Th17 cells, ROR-γt, RANK, osteoclasts, and the RANKL/OPG ratio increased and then decreased. The proportion of Treg cells and Foxp3 increased, then decreased, and increased again. Levels of RANKL and OPG increased, then decreased, then increased, and finally decreased. The Th17/Treg ratio initially decreased, then increased, and decreased again. Th17 cells were positively correlated with RANK and RANKL, the RANKL/OPG ratio, and counts of osteoclasts. Treg cells were negatively correlated with RANK expression and numbers of osteoclasts. The Th17/Treg ratio was positively correlated with RANK expression and numbers of osteoclasts.

**Conclusion:**

Under periodontal inflammation conditions, the Th17/Treg ratio might regulate orthodontic tooth movement through changing osteoclasts metabolism.

## Introduction

Periodontitis, which is a chronic and progressive disease, is one of the most common oral ailments (1). The destruction of periodontal tissue in periodontitis is characterized by periodic stages of active destruction and recovery. Patients with periodontitis exhibit displaced incisors, crowed dentition, occlusal interference, and other malocclusions (2). Orthodontic treatment can reduce periodontal trauma and promote the recovery of periodontal disease (3), and patients with periodontal disease should be treated during the recovery stage of periodontitis (4). 

 Although periodontal bacteria are the initiating factor of periodontitis, the host immune response, particularly the CD4^+^T cell-mediated cellular response, plays an important part in the development of inflammation and destruction of periodontal tissue (5). T-helper 17 (Th17) cells and regulatory T (Treg) cells are two closely related CD4^+^ T-cell subsets, and they exert reciprocal effects in the periodontal immune response (6). Th17 cells promote a pro-inflammatory state in chronic periodontitis, which potently influences osteoclast function and bone destruction (7). Treg cells play critical roles in inhibiting progressive inflammation and bone damage (8). It has been reported that the Th17/Treg balance is closely related to pathological mechanisms in periodontal disease (9).

 In previous research, we observed that the Th17/Treg ratio changes during different stages of periodontitis (10). Alveolar bone loss is a hallmark of periodontitis, even during the recovery stage of periodontitis, as the height of alveolar bone remains reduced. A major clinical challenge in the orthodontic treatment of periodontitis is preventing continuous bone loss. Therefore, it is necessary to understand how orthodontic forces induce immune factors and regulate bone resorption under conditions of periodontal inflammation. However, it is not known whether the Th17/Treg cell ratio participates in orthodontic tooth movement in periodontitis. We hypothesize that under periodontal inflammation conditions, the Th17/Treg ratio might regulate orthodontic tooth movement through changing bone metabolism. To explore these questions, we established an experimental tooth movement model in rats with periodontitis. We investigated changes in the Th17/Treg cell ratio, counts of osteoclasts, and levels of related regulatory factors during orthodontic tooth movement, and we evaluated the relationship between Th17/Treg cells, osteoclasts, and related regulatory factors.

## Materials and Methods

***Animal grouping and establishment of the periodontitis model***

Fifty-six eight-week-old male SD rats (weight:180–200 g) purchased from Vital River Laboratory Animal Technology Co., Ltd. (Beijing, China), were used in this study. All procedures and animal experiments were approved by the Animal Ethical and Welfare Committee of Beijing Stomatological Hospital & School of Stomatology, Capital Medical University (approval no. SYXK2018-0020). All rats were housed in Animal Center of Beijing Stomatological Hospital & School of Stomatology, Capital Medical University, under standard environmental conditions (12-hour light/dark cycle at 25 °C, relative humidity: 40–50%) with access to food and water *ad libitum*. Rats were randomized into two groups: a control group (24 rats) and a tooth movement during the recovery stage of the periodontitis group (RM group, 32 rats). The control group was further randomly divided into a normal control group (NC group), an active periodontitis control group (AC group), and a recovery periodontitis control group (RC group). The RM group was randomly divided into four groups: RM3 group (tooth movement for 3 days); RM7 group (tooth movement for 7 days); RM14 group (tooth movement for 14 days); and RM 21 group (tooth movement for 21 days). Each group had eight rats.

The experimental periodontitis model was established in the bilateral maxillary first molars by means of ligation and local injection with 10 µl 2 mg/ml lipopolysaccharide (LPS, Sigma, USA, L8880-10) every other day, four times (10, 11). Periodontal examinations were performed and were compared with the NC group every day. Three days after the 4th injection of LPS the periodontitis model was successfully established, which was confirmed by clinical signs, radiologic observation, and so on (10). Then rats in the AC group were euthanized, while rats in RC and RM groups were treated by removing the ligation and periodontal scaling. Periodontal examinations were performed every day after the operation to confirm when the recovery stage periodontitis models were established. When the recovery stage periodontitis was established, nickel-titanium coil springs were used in the RM groups to apply a 50-gram mesial force to move the bilateral maxillary first molars. All operations were under anesthesia to guarantee no suffering of rats.

***Flow cytometry analysis***

Fresh peripheral blood samples from abdominal aortic were collected under peritoneal anesthesia. After that, rats were euthanized without any suffering. PBMCs were isolated using a peripheral blood lymphocyte separation fluid kit (Hao Yang Biological Company, Tianjin, China, LTS1083). 

For Th17 cells analysis, PBMCs (2×10^6^/ml) were stimulated for 6 hr with Cell Stimulate Cocktail (eBioscience, USA, 00-4975-93). Cells were harvested, washed, and were surface stained with anti-rat CD4 FITC (eBioscience, USA, 11-0040-82 ) at 4 °C for 30 min, then were treated with the Fixation/Permeabilization kit (eBioscience, USA, 00-5223-56), followed by intracellular staining with anti-rat IL-17a PE (eBioscience, USA, 12-7177-81) at room temperature for 30 min. 

For Treg cell analysis, PBMCs (2×10^6^/ml) were surface-labeled with anti-rat CD4 FITC and CD25 APC (eBioscience, USA, 17-0390-82) followed by fixation and permeabilization, and intracellular staining with anti-rat Foxp3 PE (eBioscience, USA, 12-5773-80), according to the manufacturer’s instruction. 

After washing, the stained cells were evaluated by flow cytometry using an Accuri C6 instrument (BD, USA), and the results were analyzed using the FlowJo V10 software package.

***Real-time PCR***

 Four rats were randomly selected in each group and the bilateral gingival tissues surrounding the upper first molars were collected, washed in pre-chilled PBS buffer, and snap-frozen in liquid nitrogen. Total RNA was extracted from the gingival tissue of each group using the Trizol reagent (Tian Gen Biological Company, Beijing, China, DP405-02). Total RNA was then reverse-transcribed and real-time PCR was performed to assess gene expression of ROR-γt, Foxp3, and a reference gene (GAPDH), using an ABI 7500 Sequence Detection System (Applied Biosystems, USA) with a SYBR green master mix kit (Takara, Japan, RR82LR). The sequences of the synthetic oligonucleotides used for rt-PCR are given in Table 1. Relative quantification of gene expression was performed using the comparative threshold method. Changes in mRNA expression levels were calculated after normalization to GAPDH. All experiments were performed in independent triplicates.

***Histological analysis***

Harvested maxillae were fixed overnight in 4% paraformaldehyde and decalcified in 10% neutralized EDTA acid disodium salt for 8 weeks. The decalcified samples were dehydrated in graded ethanol and embedded in paraffin. Five-micrometer-thick sections were produced for immunohistochemical staining of the periodontal ligament. Histologic sections were imaged on a BX61 microscope (Olympus, Japan).

Tissue sections were deparaffinized and washed with distilled water. Pepsin was applied for 10 min for antigen retrieval. Endogenous peroxidase was blocked by incubating 10 min in 3% H_2_O_2_. Then non-specific binding sites were blocked with goat serum for 1 hr. Subsequently, samples were incubated overnight at 4 °C with anti-ROR-γt antibody (1:10 dilution; Abcam; USA, ab78007), anti-Foxp3 antibody (1:200 dilution; Abcam; USA, ab22510), anti-RANK antibody (1:50 dilution; Santa Cruz; USA, sc-59981), Anti-RANKL antibody (1:800 dilution; Abcam; USA, ab62516), and anti-OPG antibody (1:900 dilution; Abcam; USA, ab73400). After thorough rinsing, sections were incubated with the appropriate secondary antibody. After colorization with 3,3-diaminobenzidine (DAB), sections were stained by hematoxylin.

***Osteoclast TRAP staining and counting***

Tissue sections were stained with a tartrate-resistant acid phosphatase (TRAP) solution (Sigma; USA, 387A-1KT) and observed and counted using a BX61 microscope (Olympus, Japan). TRAP-positive and multinucleated cells containing three or more nuclei that were along the junction of the periodontal membrane and alveolar bone were osteoclasts. 

***Statistical analysis***

Data are presented as mean±SD. Comparisons between groups were performed by one-way analysis of variance (ANOVA). The correlation between two sets of data was analyzed by Pearson correlation analysis. If a correlation existed between two groups of variables, additional linear regression analysis was performed. Statistical analysis was performed using Statistical Package for the Social Sciences (SPSS, version 17.0; SPSS, Inc.), and* P*<0*.*05 was statistically significant.

## Results

***Flow cytometric analysis of Th17/Treg cells***

**Figure 1:**
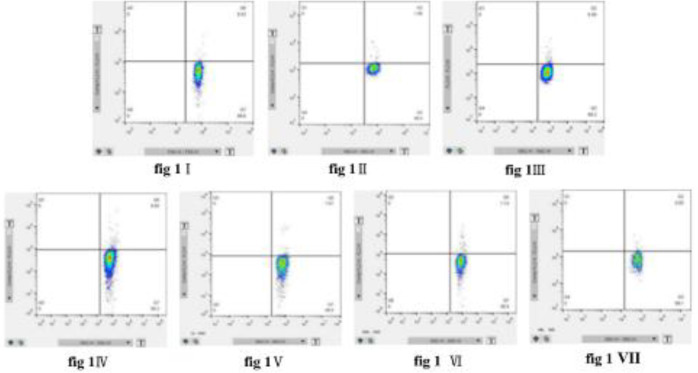
Proportion of Th17 cells in peripheral blood. Dot plot in the upper right quadrant represents CD4+IL-17+ cells (Th17 cells). (I) NC group; (II) AC group; (III) RC group; (IV) RM3 group; (V) RM7 group; (VI) RM14 group; (VII) RM21 group. The data are expressed as mean±SD of eight rats

**Figure 2:**
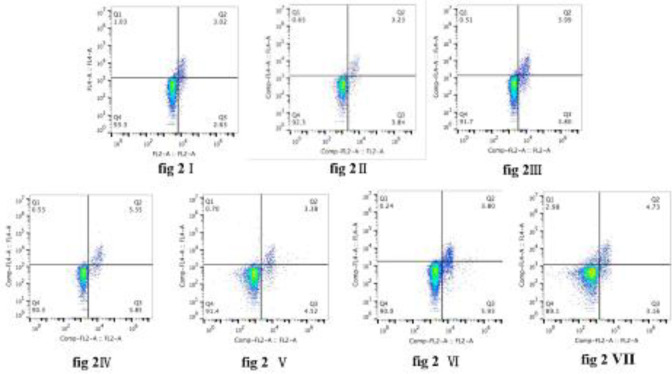
Proportion of Th17 cells in peripheral blood. Dot plot in the upper right quadrant represents CD4+CD25+ Foxp+ cells (Treg cells). (I) NC group; (II) AC group; (III) RC group; (IV) RM3 group; (V) RM7 group; (VI) RM14 group; (VII) RM21 group. The data are expressed as mean±SD of eight rats

NC: normal control; AC: active control; RC: recovery control; RM: recovery movment

**Figure 3:**
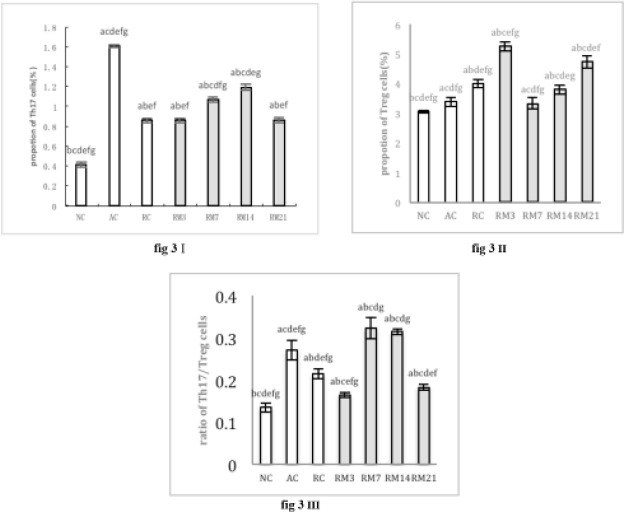
(I) Histogram expression of Th17 cells percentages. (II) Histogram expression of Treg cells percentages. (III) Histogram expression of the ratio of Th17/Treg. a: compared with NC group, *P*<0.05; b: compared with AC group, *P*<0.05; c: compared with RC group, *P*<0.05; d: compared with RM3 group, *P*<0.05; e: compared with RM7 group, *P*<0.05; f: compared with RM14 group, *P*<0.05; g: compared with RM21 group, *P*<0.05. The data are expressed as mean±SD of eight rats

NC: normal control; AC: active control; RC: recovery control; RM: recovery movment

The percentage of Th17 cells in all RM groups was higher than the NC group (*P*<0*.*05), but was significantly lower than the AC group (*P*<0*.*05; Figures 1–3). The percentage of Th17 cells increased, reaching a peak on day 14 (RM14), and then decreased (Figure 1, Figure 3I). The percentage of Treg cells initially increased, reaching a peak on day 3 (RM3), decreased to a minimum on day 7 (RM7), and then increased during orthodontic tooth movement (Figure 2, Figure 3II).

The ratio of Th17/Treg cells in all RM groups was higher than the NC group (*P*<0*.*05). Furthermore, during orthodontic tooth movement, Th17/Treg ratio first decreased on day 3 (RM3), reached a peak on day 7 (RM7), then decreased (*P*<0*.*05; Figure 3III).

***Expression of ROR-γt mRNA and Foxp3 mRNA in gingival tissue***

**Figure 4:**
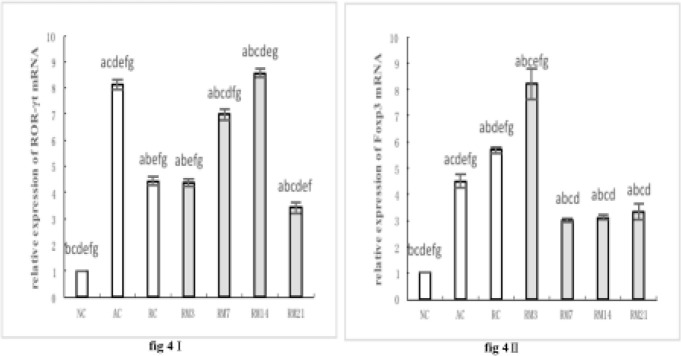
Expression of ROR-γt and Foxp3 mRNA in gingival tissues: (I) Histogram expression of ROR-γt mRNA in gingival tissues; (II) Histogram expression of Foxp3 mRNA in gingival tissues. a: compared with NC group, *P*<0.05; b: compared with AC group, *P*<0.05; c: compared with RC group, *P*<0.05; d: compared with RM3 group, *P*<0.05; e: compared with RM7 group, *P*<0.05; f: compared with RM14 group, *P*<0.05; g: compared with RM21 group, *P*<0.05. The data are expressed as mean±SD of four rats

NC: normal control; AC: active control; RC: recovery control; RM: recovery movment

The expression of ROR-γt mRNA in gingival tissue from all RM groups was higher than that of the NC group (*P*<0*.*05). Additionally, ROR-γt mRNA expression was higher in the RM14 group and lower in other RM groups compared with the AC group (*P*<0*.*05). Moreover, the expression of ROR-γt mRNA in RM groups increased to a maximum at day 14, then decreased (Figure 4I).

The expression of Foxp3 mRNA in all RM groups was higher than that of the NC group (*P*<0*.*05). Additionally, Foxp3 mRNA expression was higher in the RM3 group and lower in other RM groups compared with the AC group (*P*<0*.*05). Moreover, the expression of Foxp3 mRNA in RM groups reached a maximum on day 3, then decreased to a minimum on day 7, and increased gradually over the course of the study (Figure 4II)

**Figure 5:**
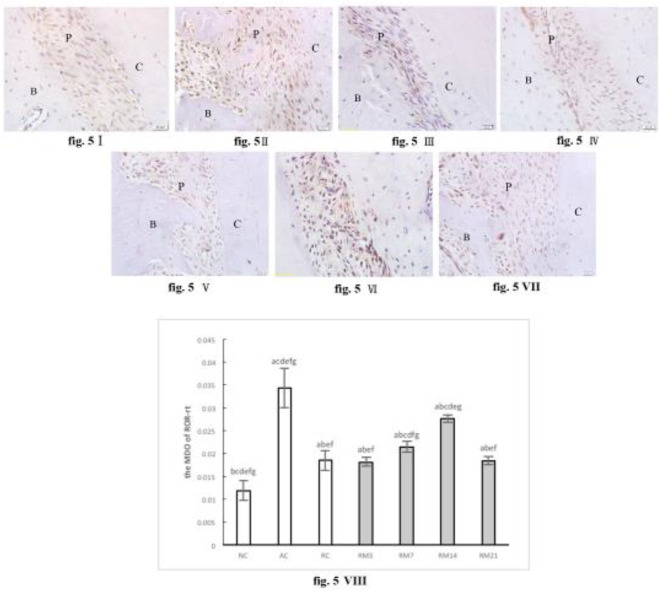
Immunohistochemically stained ROR-γt in periodontium (40× magnification). (I) NC group; (II) AC group; (III) RC group; (IV) RM3 group; (V) RM7 group; (VI) RM14 group; (VII) RM21 group. (B: alveolar bone, C: cementum, P: periodontal ligaments). (VIII) The mean optical density (MOD) of ROR-γt in periodontium (a: compared with NC group, *P*<0.05; b: compared with AC group, *P*<0.05; c: compared with RC group, *P*<0.05; d: compared with RM3 group, P<0.05; e: compared with RM7 group, *P*<0.05; f: compared with RM14 group, *P*<0.05; g: compared with RM21 group, *P*<0.05). The data are expressed as mean±SD of four rats

NC: normal control; AC: active control; RC: recovery control; RM: recovery movment

Immunohistochemistry analysis of protein expression in periodontal ligaments showed that ROR-γt staining was detected in the periodontium from AC, RC, and RM groups, but was barely detected in the NC group. The expression of ROR-γt in all RM groups was higher than that of the NC group and lower than in the AC group. In RM groups, ROR-γt protein expression exhibited an increasing trend initially, reaching a peak on day 14 (RM14 group) (Figure 5).

**Figure 6:**
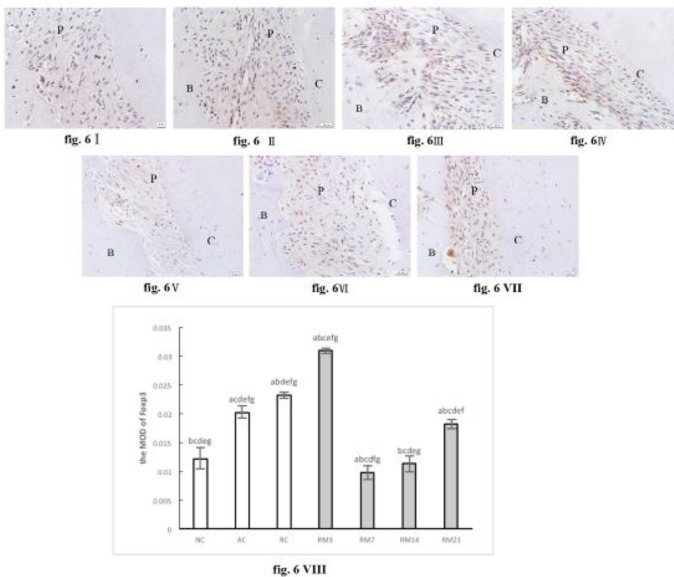
Immunohistochemically stained Foxp3 in periodontium (40× magnification). (I) NC group; (II) AC group; (III) RC group; (IV) RM3 group; (V) RM7 group; (VI) RM14 group; (VII) RM21 group. (B: alveolar bone, C: cementum, P: periodontal ligaments). (VIII) The mean optical density (MOD) of Foxp3 in periodontium (a: compared with NC group, *P*<0.05; b: compared with AC group, *P*<0.05; c: compared with RC group, *P*<0.05; d: compared with RM3 group, *P*<0.05; e: compared with RM7 group, *P*<0.05; f: compared with RM14 group, *P*<0.05; g: compared with RM21 group, *P*<0.05). The data are expressed as mean±SD of four rats

NC: normal control; AC: active control; RC: recovery control; RM: recovery movment

Foxp3 staining was observed along the periodontal ligament tissue in AC, RC, and RM groups, and was rarely detected in the NC group. Moreover, the expression of Foxp3 increased to a peak on day 3 (RM3 group), then reached a minimum on day 7 (RM7 group), and again increased gradually over the course of the experiment (Figure 6).

**Figure 7:**
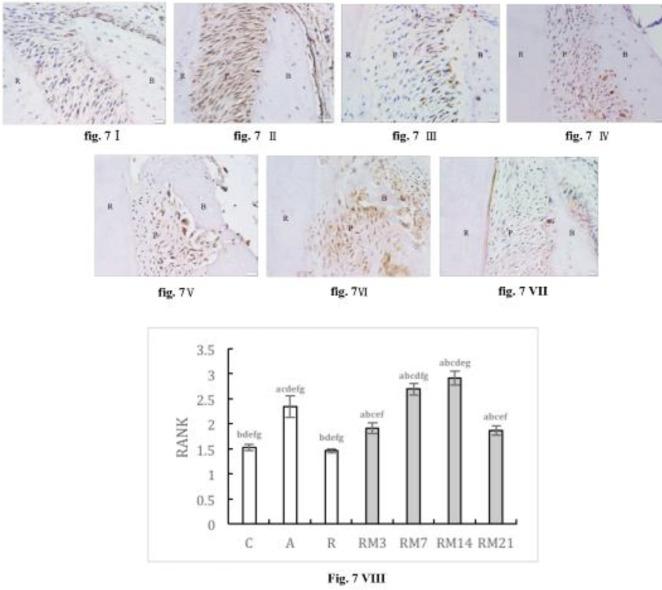
Immunohistochemically stained RANK in periodontium (40× magnification). (I) NC group; (II) AC group; (III) RC group; (IV) RM3 group; (V) RM7 group; (VI) RM14 group; (VII) RM21 group. (B: alveolar bone, C: cementum, P: periodontal ligaments). (VIII) The mean optical density (MOD) of Foxp3 in periodontium ( a: compared with NC group, *P*<0.05; b: compared with AC group, *P*<0.05; c: compared with RC group, *P*<0.05; d: compared with RM3 group, *P*<0.05; e: compared with RM7 group, *P*<0.05; f: compared with RM14 group, *P*<0.05; g: compared with RM21 group, *P*<0.05). The data are expressed as mean±SD

NC: normal control; AC: active control; RC: recovery control; RM: recovery movment

**Figure 8:**
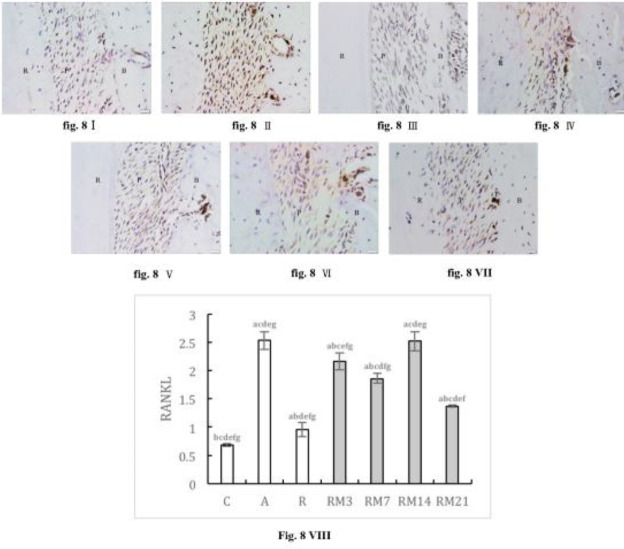
Immunohistochemically stained RANKL in periodontium (40× magnification). (I) NC group; (II) AC group; (III) RC group; (IV) RM3 group; (V) RM7 group; (VI) RM14 group; (VII) RM21 group. (B: alveolar bone, C: cementum, P: periodontal ligaments). (VIII) The mean optical density (MOD) of Foxp3 in periodontium ( a: compared with NC group, *P*<0.05; b: compared with AC group, *P*<0.05; c: compared with RC group, *P*<0.05; d: compared with RM3 group, *P*<0.05; e: compared with RM7 group, *P*<0.05; f: compared with RM14 group, *P*<0.05; g: compared with RM21 group, *P*<0.05). The data are expressed as mean±SD

NC: normal control; AC: active control; RC: recovery control; RM: recovery movment

The expression of RANK and RANKL was increased in AC, RC, and RM groups, and was higher in all RM groups compared with the RC group. During orthodontic tooth movement, both RANK and RANKL expression initially increased, then decreased, and reached a peak on day 14. At peak expression, RANK in the RM14 group was higher than that in the AC group, however, RANKL expression was not different from the AC group (Figures 7-8).

**Figure 9:**
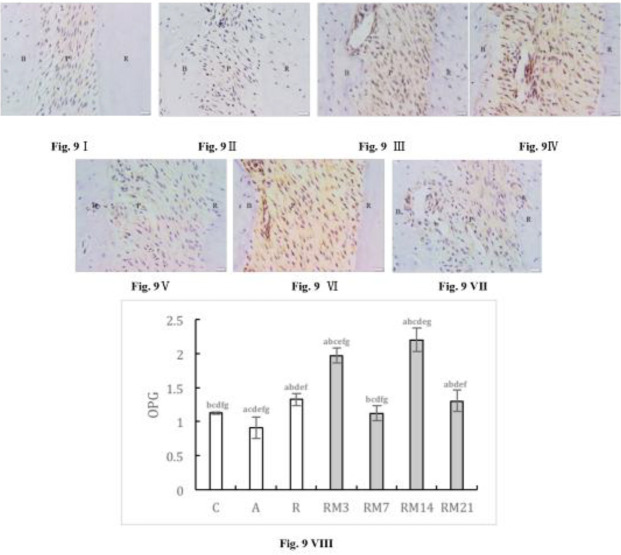
Immunohistochemically stained OPG in periodontium (40× magnification). (I) NC group; (II) AC group; (III) RC group; (IV) RM3 group; (V) RM7 group; (VI) RM14 group; (VII) RM21 group. (B: alveolar bone, C: cementum, P: periodontal ligaments). (VIII) The mean optical density (MOD) of Foxp3 in periodontium ( a: compared with NC group, *P*<0.05; b: compared with AC group, *P*<0.05; c: compared with RC group, *P*<0.05; d: compared with RM3 group, *P*<0.05; e: compared with RM7 group, *P*<0.05; f: compared with RM14 group, *P*<0.05; g: compared with RM21 group, *P*<0.05). The data are expressed as mean±SD

NC: normal control; AC: active control; RC: recovery control; RM: recovery movment

OPG expression in the AC group was lower than that in the NC group, RC group, and all RM groups. During orthodontic tooth movement, OPG initially increased, then decreased, then increased, and finally decreased. In RM groups, OPG expression peaked on day 14 and was higher than in the RC group. On day 7, OPG expression fell to a minimum value, and there was no difference compared with the NC group (Figure 9).

**Figure 10:**
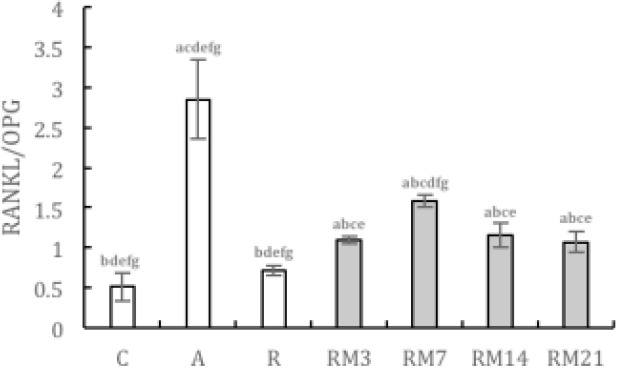
Histogram expression of the ratio of RANKL/OPG. (C) NC group; (A) AC group; (R) RC group; (RM3) RM3 group; (RM7) RM7 group; (RM14) RM14 group; (RM21) RM21 group. ( a: compared with NC group, *P*<0.05; b: compared with AC group, *P*<0.05; c: compared with RC group, *P*<0.05; d: compared with RM3 group, *P*<0.05; e: compared with RM7 group, *P*<0.05; f: compared with RM14 group, *P*<0.05; g: compared with RM21 group, *P*<0.05). The data are expressed as mean±SD

NC: normal control; AC: active control; RC: recovery control; RM: recovery movment

The ratio of RANKL/OPG in the AC group was higher than in NC and RC groups. However, during orthodontic tooth movement in rats with periodontitis, the ratio of RANKL/OPG first increased and then decreased, reaching a peak on day 7, and was lower than that in the AC group (Figure 10).

***Osteoclasts TRAP staining and counting***

**Figure 11:**
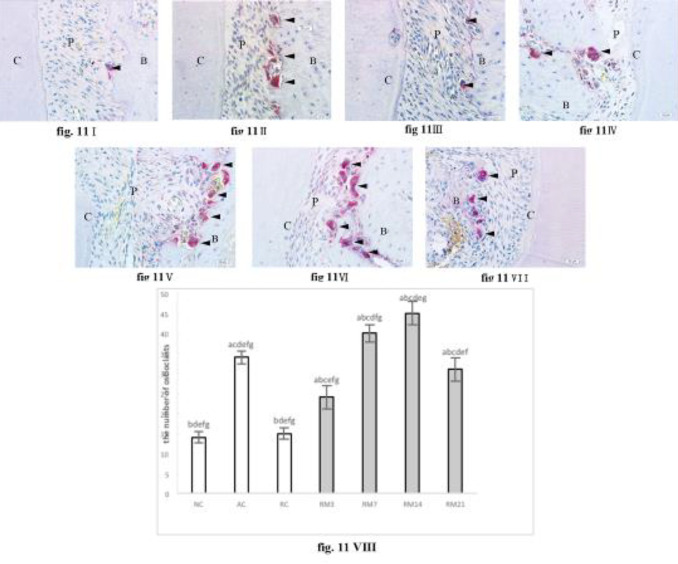
Osteoclasts TRAP staining (40× magnification). (I) NC group; (II) AC group; (III) RC group; (IV) RM3 group; (V) RM7 group; (VI) RM14 group; (VII) RM21 group; (VIII) Osteoclast counting (a: compared with NC group,* P*<0.05; b: compared with AC group, * P*<0.05; c: compared with RC group, *P*<0.05; d: compared with RM3 group, * P*<0.05; e: compared with RM7 group, * P*<0.05; f: compared with RM14 group, *P*<0.05; g: compared with RM21 group, *P*<0.05). The data are expressed as mean±SD of four rats

NC: normal control; AC: active control; RC: recovery control; RM: recovery movment

Osteoclasts were barely detected in the NC group, and osteoclast counts were higher in the AC group than that in NC and RC groups (*P*<0*.*05; Figure 11). During orthodontic tooth movement, osteoclast counts first increased, then decreased, reaching peak counts on day 14, and were higher than that in the AC group (*P*<0*.*05; Figure 11).

***Correlation and regression analysis***

Pearson correlation analysis and regression analysis were performed between the proportion of Th17 cells, Treg cells, and the ratio of Th17/Treg cells and levels of osteoclast related factors during orthodontic tooth movement in rats with periodontitis. The proportion of Th17 cells was positively correlated with RANK expression, RANKL expression, the RANKL/OPG ratio, and osteoclast counting. The proportion of Treg cells was negatively correlated with RANK expression and osteoclast counting, and there were linear relationships between these factors. The Th17/Treg ratio was positively correlated with RANK expression and osteoclast counting, and there was a linear relationship between them. There was no correlation found between the Th17/Treg ratio and RANKL/OPG ratio (Table 2).

**Table 1:** The sequences of the synthetic oligonucleotides used for RT-PCR

Molecule	Sequence (5’-3’)
ROR-γt	Forward: ATGGAAGTCGTCCTCGTCAGAAT
	Reverse: ATGCTCCACTCTCCTCTTTTCTTG
Foxp3	Forward: CCTATGCCACCCTCATCCGA
	Reverse: CTCTCCACTCGCACAAAGCACT
GAPDH	Forward: TTCCTACCCCCAATGTATCCG
	Reverse: CCACCCTGTTGCTGTAGCCATA

NC: normal control; AC: active control; RC: recovery control; RM: recovery movment

## Discussion

Although combined periodontal-orthodontic therapy is regularly used to treat periodontitis patients with malocclusions, whether the mechanical forces involved increase the risk of bone absorption remains an open question(3, 12). Chronic periodontitis is characterized by loss of periodontal attachment and alveolar bone absorption and alternately appears in the active stage and recovery stage. Th17 cells and Treg cells play important roles in regulating periodontitis. Th17 cells exhibit high pro-inflammatory activity in periodontal tissues, and Treg cells play an important role in reducing the inflammatory response and bone absorption(7, 13-15). It has been reported that an imbalance in the Th17/Treg ratio could lead to the occurrence and development of periodontitis (16,17) and that periodontitis could result in an increase in Th17 cells and a decrease in Treg cells (18). It is widely accepted that the optimal opportunity for orthodontic intervention in patients with periodontitis is during the recovery stage of periodontitis (4). However, whether and how orthodontic force induces Th17/Treg cells to regulate orthodontic tooth movement in periodontitis remains unknown.

In this study, we show that periodontal inflammation can increase the proportion of Th17 cells, Treg cells, and the Th17/Treg ratio, supporting the evidence linking Th17/Treg cells and periodontitis. Furthermore, in our model in which teeth were moved during the recovery stage of periodontitis, we found that orthodontic force caused significant changes in the Th17 and Treg cell proportions. The proportion of Th17 cells in peripheral blood, and the mRNA expression of the transcription factor ROR-γt in gingival tissues, increased to a peak at day 14, then decreased. The proportion of Treg cells and the mRNA expression of the related transcription factor Foxp3 initially increased to a maximum on day 3, then decreased to a minimum on day 7, and again increased. Meanwhile, the Th17/Treg ratio reached a peak on day 7 to day 14 (no difference between days 7 and 14), then decreased during orthodontic tooth movement under inflammatory periodontal conditions. We surmise that the reason for this might be that orthodontic tooth movement is related to stress responses triggered by the existing inflammatory environment. Inflammation is an effective defense mechanism when the body faces external force stimuli. Therefore, in the initial stages of orthodontic treatment, the proinflammatory index may be higher, but with the passage of time, the body gradually adapts and the anti-inflammatory index begins to work, controlling the inflammation and reaching a relatively normal and stable balance (19). Moreover, the proportion of Th17 cells and the Th17/Treg ratio was lower in each RM group than that in the AC group, indicating that when active periodontal inflammation enters the recovery stage, although orthodontic force may increase the differentiation of Th17 cells, the inhibitory action of Treg cells is also activated by orthodontic force early in the recovery stages of periodontitis. This balance may take an important part in protecting periodontal tissue from progressive damage during the recovery stage of periodontitis.

We also evaluated changes of osteoclasts and the osteoclast-related regulatory factors RANK, RANKL, and OPG, during orthodontic tooth movement with periodontitis. We found that orthodontic force also influences osteoclasts and their related regulatory factors, which can influence orthodontic bone remodeling in periodontitis. During orthodontic tooth movement, RANK expression, the RANKL/OPG ratio, and counts of osteoclasts showed similar tendencies to initially increase, then to decrease. However, RANKL and OPG expression were first increased and then decreased, and increased again and finally decreased. These were in line with a report by Okamoto* et al*. (20), in which it was shown that during orthodontic treatment with periodontitis, PGE_2_ is increased and that PGE_2_ can inhibit RANKL release from fibroblasts by blocking the Ras/Raf/ERK signaling pathway, which is not only activated by orthodontic force but can also promote OPG release, in turn inhibiting the differentiation and maturation of osteoclasts.

Our results showed that orthodontic force under periodontal inflammation not only increases the proportions of Th17 and Treg cells but also increases numbers of osteoclasts and induces changes in the expression of related regulatory factors. In order to detect whether and how Th17/Treg cells take part in regulating osteoclasts, we analyzed correlations between Th17/Treg cells, osteoclasts, and related regulatory factors. We found that Th17 cells were significantly correlated with RANK expression, RANKL expression, the RANKL/OPG ratio, and numbers of osteoclasts, but were not correlated with OPG expression. This suggests that Th17 cells promote osteoclast formation by increasing RANK and RANKL expression. This is in agreement with studies that have demonstrated that Th17 cells can directly secrete RANKL, or can secrete IL-17 to promote RANKL expression, and inhibit OPG expression, thus increasing the RANKL/OPG ratio to promote osteoclast formation and function(21). Th17 cells were significantly correlated with RANK expression during orthodontic tooth movement with periodontitis; this may be because Th17 cells can activate the production of M-CSF by the release of PGE_2 _(22). Th17 cells can interact with M-CSF on monocytes to induce its differentiation into osteoclasts (23). RANK is a transmembrane protein located in mononuclear macrophage and osteoclast cell lines (24), so its expression might increase along with increased populations of osteoclasts. Our findings suggest that Treg cells are negatively correlated with RANK expression and numbers of osteoclasts, but did not display any significant relationship with other factors. This relationship may be related to regulation of the RANK/RANKL/OPG system by cytokines secreted by Treg cells. TGF-β, IL-10, and CTLA-4 secreted by Treg cells can promote OPG expression and can inhibit RANKL expression, thus reducing the RANKL/OPG ratio and inhibiting osteoclast generation and bone resorption (25, 26).

In addition, CTLA-4 on the surface of Treg cells can directly contact osteoclast precursor cells and inhibit their differentiation into mature osteoclasts (27); this might also decrease RANK expression. We suggest that the inhibitory effects of Treg cells on RANK and osteoclasts might be a the critical mechanism to protect alveolar bone from excessive resorption during orthodontic tooth movement under conditions of periodontal inflammation. 

We also found that the Th17/Treg ratio was significantly correlated with RANK expression and numbers of osteoclasts. It can be inferred that the relationship between the Th17/Treg ratio and osteoclast-related factors is complicated during orthodontic tooth movement with periodontitis and that Treg cells might be key mediators that influence the process of orthodontic tooth movement. Additionally, the Th17/Treg ratio might affect the formation and function of osteoclasts in other ways beyond regulating the RANKL/OPG ratio. Adding to the complexity of these interactions, the RANK/RANKL/OPG system can also influence the Th17/Treg ratio. However, the limitation of this study is that the mechanisms of how the Th17/Treg ratio regulates bone metabolism have not been investigated yet. That would be our future study.

## Conclusion

 We demonstrated that orthodontic force induces Th17 and Treg cells, osteoclasts, and changes the expression of osteoclast-related factors under conditions of periodontal inflammation. Our data indicate that Th17 and Treg cells participate in regulating orthodontic tooth movement. Changes in the Th17/Treg ratio exhibited significant correlation with osteoclasts and osteoclast-related regulatory factors. Therefore, we concluded that under periodontal inflammation conditions, the Th17/Treg ratio might regulate orthodontic tooth movement through changing osteoclasts metabolism. 

## Founding Statement

This research is supported by the Beijing Natural Science Foundation (award number 7182062).
